# Influence of misorientation angle and local dislocation density on β-phase distribution in Al 5xxx alloys

**DOI:** 10.1038/s41598-022-05948-8

**Published:** 2022-02-02

**Authors:** Jahnavi Desai Choundraj, Josh Kacher

**Affiliations:** School of Materials Science and Engineering, Georgia Institute of Technology, Atlanta, GA USA

**Keywords:** Materials science, Metals and alloys

## Abstract

Al–Mg alloys undergo sensitization when exposed to elevated temperatures, making them susceptible to intergranular corrosion and stress corrosion cracking. Most of the existing research on microstructure effects on sensitization is centered on the effect of intrinsic grain boundary characteristics such as misorientation angle and coincident site lattice (CSL) values. Very few studies have systematically investigated the influence of extrinsic characteristics such as dislocation density. In this paper, the influence of local microstructure characteristics on the sensitization susceptibility of AA5456 was investigated using in situ optical microscopy corrosion experiments and electron back scattering diffraction analysis. The results show a clear trend between the local geometrically necessary dislocation (GND) density and β phase precipitation, with higher GND densities correlating with higher rates sensitized boundaries. This trend held true even for low angle grain boundaries. These results demonstrate the importance of considering factors beyond grain boundary characteristics in determining susceptibility to sensitization.

## Introduction

5xxx series Al alloys are non-heat treatable, moderate strength wrought alloys with 3–5% Mg. These alloys are widely used for marine, automotive, and military applications due to their high strength-to-weight ratio, mechanical properties, and corrosion resistance^1^. However, at moderate and high temperatures (50–250 °C) these alloys are vulnerable to sensitization, which is precipitation of β phase (Al_3_Mg_2_) preferentially at grain boundaries (GBs)^2^. This β phase is more electrochemically active compared to the rest of the Al matrix^3–5^. As a result, the β phase is preferentially dissolved when the alloys are exposed to corrosive environments such as salt water, making them highly susceptible to intergranular corrosion (IGC) and to some extent stress corrosion cracking (SCC)^3,6,7^. Multiple reports have correlated high degrees of sensitization values with increased corrosion fatigue crack growth rates^8^ and the intergranular β phase coverage to intergranular stress corrosion cracking (IGSCC) susceptibility, which is the nucleation and growth of cracks along the corroded grain boundaries in the presence of stress, in Al–Mg alloys^3,4,6,7,9^, motivating the search for sensitization-resistant Al alloys.

Studies on β phase formation have shown that super saturated solid solution of Mg is the starting point, from which GP zones (Guinier Preston Zones, also known as δ″) consisting of Mg rich clusters form. These are observed at relatively low temperatures up to the critical temperature range of 45–50 °C^10,11^. Once the critical temperature is reached, these clusters transform to β″ spherical particles with a composition of Al_3_Mg^12,13^. The GP zones and β″ particles dissolve and β′ precipitates and vacancy voids/dislocation loops form at grain boundaries during annealing. This formation begins at 100 °C and accelerates at 150 °C^14^. At temperatures above 200 °C, the stable equilibrium β phase forms from β′ and above 250 °C β phase forms from the direct decomposition of Al matrix super saturated solid solution^2,10,11^. A recent study reported the existence of stable β at higher temperatures (325 °C)^15^. The precipitate formation was observed mainly at the grain boundaries and defects in the material^14,16^. However, there exists a lack of information on which microstructural features influence the β precipitation during sensitization.

Several studies have been conducted to understand the influence of grain boundary character, especially the misorientation angle, on β precipitation. Work by Davenport et al. on sensitized AA5182 alloy reported that low angle grain boundaries are immune to sensitization as determined by a H_3_PO_4_ etch^17^. Similarly, work by Kaigorodova suggested that β phase growth is more favorable on high angle grain boundaries compared to low angle grain boundaries^18^. While most studies suggest that low angle boundaries are immune to precipitation due to their low interfacial energies^17–19^, Scotto D’Antuono et al.^20^ showed that precipitation exists at grain boundaries with low angle misorientation. Accelerated in situ transmission electron microscopy (TEM) heating experiments conducted on AA5456 H116 alloy revealed β precipitates on both on low and high angle grain boundaries^21^. A recent study by Zhang et al. comparing the continuity of β phase on high angle and low angle grain boundaries found that high angle grain boundaries have higher continuity in precipitation, making them more vulnerable to IGC^22^. These studies suggest that grain boundary misorientation angle alone does not dictate susceptibility to β phase formation, prompting the investigation of additional factors such as dislocation density and proximity to intermetallic particles.

Beyond grain boundary characteristics, there have been a few studies on the effect of dislocation density on β precipitation^4,23–27^. Scotto D’Antuono et al. investigated the formation and growth of β precipitates in AA5456 using in situ TEM annealing experiments at 300 °C. An increase in growth rate of the precipitates was reported due to pipe diffusion through dislocations near the grain boundaries^23^. This study also reported favorable heterogeneous nucleation of precipitates at Mn-rich particles. A similar effect was observed in a nanocrystalline Al-7.5% Mg synthesized by cryomilling and hot pressing. Grain boundaries enriched with Mg were observed under high resolution TEM, which was attributed to the high concentration of dislocations closer to grain boundaries providing diffusion channels and accelerating the sensitization^24,25^. Gronksy and Furrer reported that the presence of dislocations would dominate the precipitation reaction under certain conditions^26^. These studies have in general relied on a relatively small number of grain boundaries, with questions remaining about the statistical importance of dislocation density on sensitization susceptibility. A study performed by Tan and Allen^4^ used a thermomechanical treatment to enhance the corrosion resistance of AA5083 alloy and found that increases in the global dislocation density and low angle grain boundaries lead to better corrosion resistance. However, this study did not investigate the influence of the local microstructure state on IGC susceptibility.

In the current study, the combined influence of dislocation density and grain boundary misorientation angle on β precipitation was investigated using a correlative optical microscopy/electron backscatter diffraction (EBSD)-based approach. Previous work has shown that it is possible to quantify correlative relationship using EBSD to understand the effects of grain boundaries and secondary phase particles on dislocation accumulation^28^. The current study uses a similar correlative microscopy approach to rapidly characterize hundreds of grain boundaries in terms of their misorientation angle, surrounding defect distribution, and prevalence of β phase precipitates and to establish quantitative correlations between them.

## Materials and methods

### Sample preparation

Commercial Al alloy 5456 H116 samples (obtained from McMaster-Carr) of dimension 1 × 1 × 0.6 cm (L × W × H) were used for carrying out the experiments in this study. The composition of this alloy is given in Table 1. The samples were cut directly from 0.6 cm thickness sheet after which they were sensitized by heat treating at 150 °C for 72 h in a furnace followed by air quenching. The samples were then polished using diamond suspensions down to 1 µm followed by colloidal silica suspension (0.05 µm). To remove the mechanical polishing-induced damaged layer for EBSD analysis, the polished samples were ion milled at an accelerating voltage of 3 kV, beam incidence angle of 60°, and 1 mm offset for 5 min under flat milling conditions. The ion milled samples were then indented to create fiduciary markers using Vickers microhardness tester, using a force of 300 gf to generate an indented area of 250 × 250 µm.

**Table 1:** Elemental composition of AA5456 alloy.

Element	Al	Mg	Mn	Fe	Si	Cr	Zn	Ti	Cu
Wt.%	92–94.8	4.7–5.5	0.5–1	0–0.4	0–0.25	0.05–0.2	0–0.25	0–0.2	0–0.1

**Figure 1:**
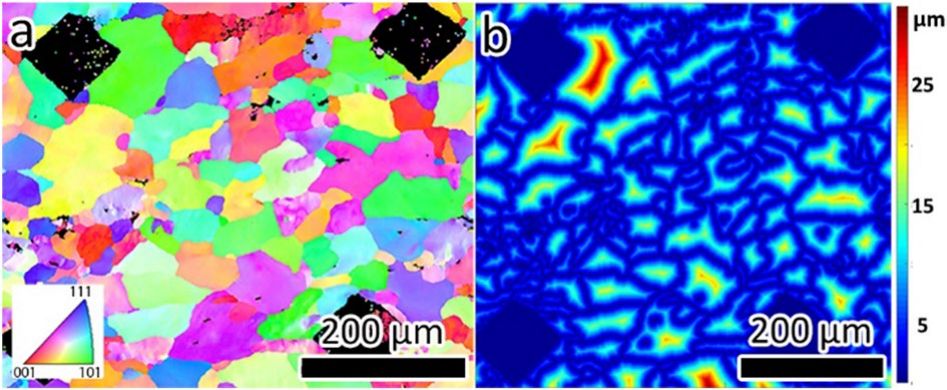
(**a**) IPF map with grains color coded based on the crystal orientation. Data points with confidence less than 0.1, as defined by the EDAX/TSL analysis software, are colored black. (**b**) Distance to grain boundary map used for calculation of GND density near the grain boundaries.

The crystallographic microstructural information of the samples was obtained by EBSD using a Tescan MIRA scanning electron microscope (SEM) at an accelerating voltage of 20 kV and a step size of 0.5 µm. This step size was chosen to get an accurate estimate of the local GND (geometrically necessary dislocation) density while still being able to characterize relatively large areas. Figure 1a shows an inverse pole figure (IPF) map generated from the EBSD scan.

### In situ optical microscopy experiments

To directly observe dissolution of the β phase, the sensitized and polished samples were placed in a petri-dish under an optical microscope and the long transverse face of the samples were exposed to a thin layer of 1% phosphoric acid solution. This acid attacks the β phase of the material preferentially and causes intergranular corrosion (as β is the only anodic phase present on the grain boundaries) and has been used traditionally to reveal the locations of β^2,3,17,19,29^. The experiments were carried out for 40 min during which time corrosion events were recorded using an optical microscope with 20× magnification using a real-time video digital microscope. Still shots of the acid exposed samples were taken using 40× magnification to resolve the corroded grain boundaries more clearly.

### Microstructure and GB characterization

As mentioned earlier, phosphoric acid was used to etch the β phase specifically, to locate the β particles in the matrix. Thus, the etched spots visible in the optical microscopy images correspond to the distribution of β phase in the material. These images extracted from the recorded videos were used to identify the corroded grain boundaries and to create corrosion maps spatially aligned with the EBSD data, using the EBSD image quality (IQ) maps and fiducial markers to manually align images from the two sources. With this spatial alignment, correlative relationships were established between grain boundary characteristics, including the surrounding environment, and likelihood of grain boundary attack. Detailed analysis of the data was then used to understand the influence of microstructural parameters on β phase precipitation.

The microstructural parameters considered in this research included misorientation angle and GND density. This information was obtained from EBSD characterization. Each grain in the EBSD scans was assigned a unique numerical identifier. The grain boundaries could then be uniquely identified based off of the identities of the grains they separated, and the grain boundary information (misorientation angle and GND density) was calculated for each boundary. GND density was calculated using the Nye’s tensor-based approach^30–32^. In this approach, the curvature tensor is first calculated by taking the curl of the orientation field. Assuming that elastic strain gradients are negligible, this tensor can be related to Nye’s dislocation density tensor using the relationship developed by Kroner^33^.

1 $${\alpha }_{ik}= {\kappa }_{ki}- {\delta }_{ki}-{\epsilon }_{klj}\frac{\partial {\varepsilon }_{ij}^{el}}{\partial {x}_{l}}$$

and the curvature tensor, к, is given by

2 $${\kappa }_{ki}= \frac{\partial {\theta }_{k}}{\partial {x}_{i}}$$

where ${\alpha }_{ik}$ is the Nye’s dislocation density tensor, where rows correspond to the Burger’s vector direction and columns correspond to the line direction. ${\epsilon }_{klj}$ is the Levi–Civita permutation symbol, ${\varepsilon }_{ij}^{el}$ is the elastic strain tensor and ${\theta }_{k}$ is the rotation vector.

As EBSD scans are limited to two dimensions, only five components of the dislocation density tensor can be accessed directly. The other components are assumed to be the average of the known components and the GND density is taken to be the L1 norm of Nye’s tensor, based off of the work by Ruggles et al.^32^.

To check the accuracy of the GND calculations, following equation was used to estimate the limiting dislocation density resolution^31^:

3 $${\rho }_{min}^{GND}= \frac{{\theta }_{min}}{Lb}$$

where ${\rho }_{min}^{GND}$ is the limiting dislocation density resolution or expected noise level, ${\theta }_{min}$ is the chosen angular resolution, L is step size of the scan and b is the magnitude of Burger’s vector of the material used. Using the above Eq. (3) and a step size of 0.5 µm, b value for aluminum as 2.86 × 10^−10^ m and 0.5° of theta, the limiting resolution for dislocation density was found to be 6.08 × 10^13^ m^−2^^34^. Most of the GND values calculated are well above this limiting value, suggesting that the GND values are accurate.

The GND density was calculated at every point located within 0.5 µm of a grain boundary. This is based on the limitation of the equipment resolution and the step size used in our scans, which is 0.5 µm. An average value was then calculated and taken to represent the mean GND density of that grain boundary^28,30,35–37^ (Fig. 1b shows the distance-to-grain boundary map calculated from the EBSD data).

To calculate the grain boundary misorientation angle, Bunge’s orientation matrix (g) was initially calculated for two grains separated by the grain boundary using the Euler angles measured by EBSD. The misorientation matrix between for these two grains (say A, B) was then obtained from^38^;

$$\Delta {\mathrm{g}}_{AB}={\mathrm{g}}_{A}{\mathrm{g}}_{B}^{T}$$

where *T* denotes the transpose of the orientation matrix, which is also equivalent to the inverse. The misorientation angle was then obtained using the following equation:

$$\theta ={\mathrm{cos}}^{-1}\left(\frac{Tr(\Delta {\mathrm{g}}_{AB})}{2}\right)$$

Here, *Tr* represents the trace. Misorientation angle was calculated for each of the cubic symmetry operators applied to the matrix $\Delta {\mathrm{g}}_{AB},$ and the minimum angle was taken as the misorientation angle.

Thus, the proximal GND density was calculated for each grain boundary and the grain boundary misorientation information was obtained. This information was used to define the grain boundary state and to perform qualitative and quantitative analysis.

IGC susceptibility in Al alloys is traditionally assessed using NAMLT (nitric acid mass loss test) values. The IGC spreading observed in this experiment is attributed to its high NAMLT value, which was found by the ASTM G67-18 test to be 75 mg cm^−2^. This is in accordance with the earlier reports which suggest that only highly sensitized conditions lead to IGC spreading due to the formation of a large network of nearly continuous β phase covered grain boundaries in close radial proximity^39^.

## Results

Low Ʃ boundaries were investigated, but did not show any correlation with β precipitation and so are not further discussed in this paper. Moreover, unlike Ni and steel^40,41^, bulk Al does not form coherent twin boundaries or low Ʃ boundaries readily via grain boundary engineering, making this an unviable approach for reducing susceptibility to sensitization and IGC in these alloys.

### In situ optical microscopy

**Figure 2:**
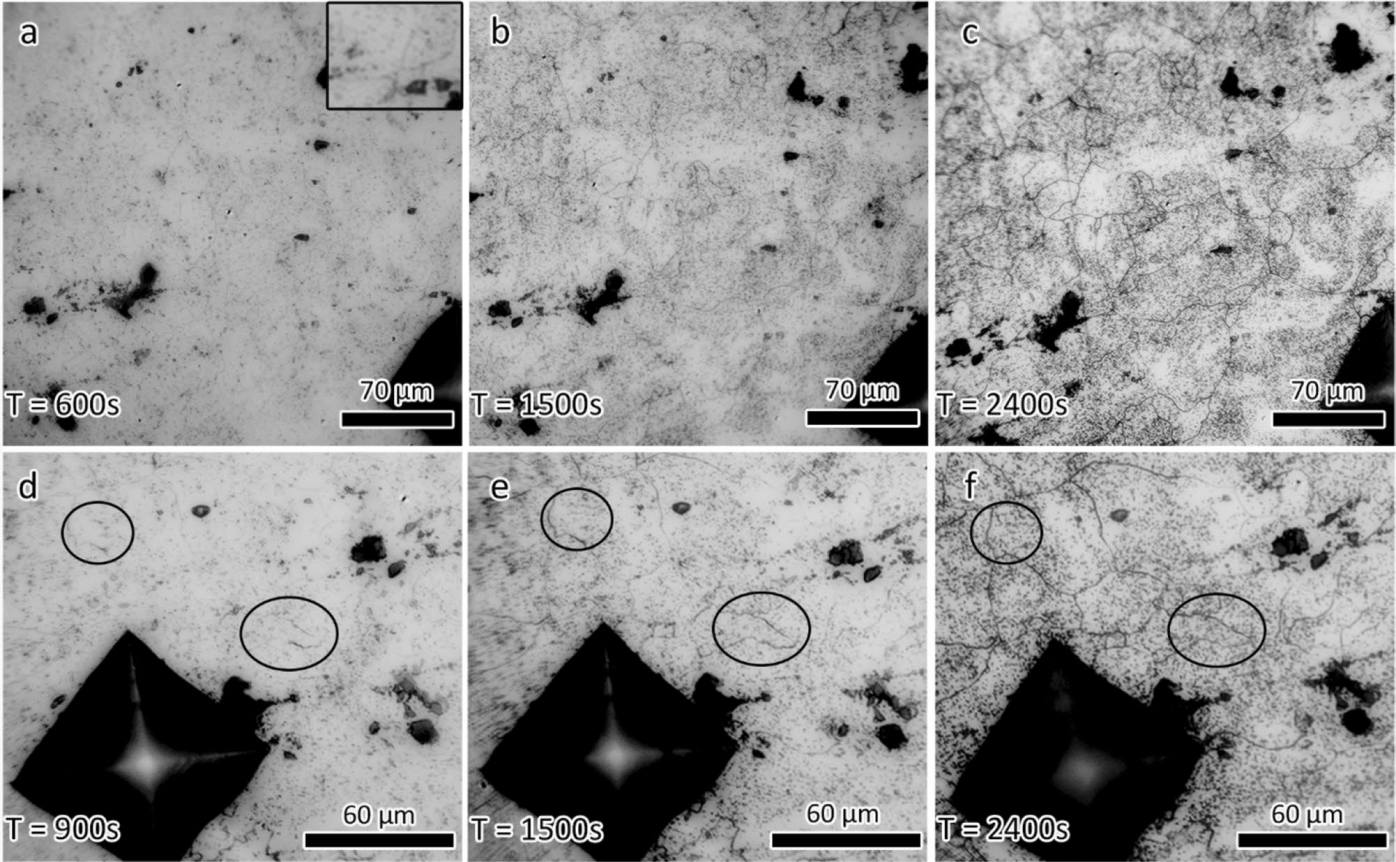
(**a**)–(**c**) Optical images taken during the in situ optical microscopy corrosion experiment. Inset in (**a**) highlights a grain boundary attacked at the earliest stage of corrosion. (**d**)–(**f**) Different region on the same sample. Circles highlight corrosion progression of partially corroded GBs to fully corroded GBs. Experiment time is given in each panel.

Figure 2 shows images of the corroded samples at different stages of the in situ optical microscopy experiments. The attacked boundaries appear dark in the optical images. As can be seen, the fraction of corroded boundaries increases as the experiment time increases (Fig. 2a–c). The first appearance of grain boundary corrosion was seen after approximately 500–600 s of acid exposure. Corroded grain boundaries were manually identified at different stages of the testing to determine the fraction of attacked grain boundaries as a function of time. At the earliest stage of corrosive attack, after 600 s of exposure, approximately 3% of the total grain boundaries were corroded. This increased to approximately 6% of the boundaries being attacked at the 1200 s point, or roughly a doubling in the number of attacked grain boundaries as compared to the 600 s point. After 2400 s of exposure to the acid, 96% of the grain boundaries were attacked, showing a significant increase in the rate of attack as compared to the first 1200 s of the experiment. This increased attack included grain boundaries that were partially corroded at the beginning of the experiment, but fully corroded by the end (Fig. 2d–f).

Post-acquisition, the optical images were aligned with the EBSD maps, facilitating direct correlations between the attacked boundaries and microstructural characteristics. These correlations are highlighted in the following sections, dividing the analysis between the most susceptible boundaries (*i.e.* those that were attacked in the early stages of the experiment) and the most resistant boundaries (*i.e.* those that remained unattacked at the end of the experiment).

### Early stage microstructure effects

**Figure 3:**
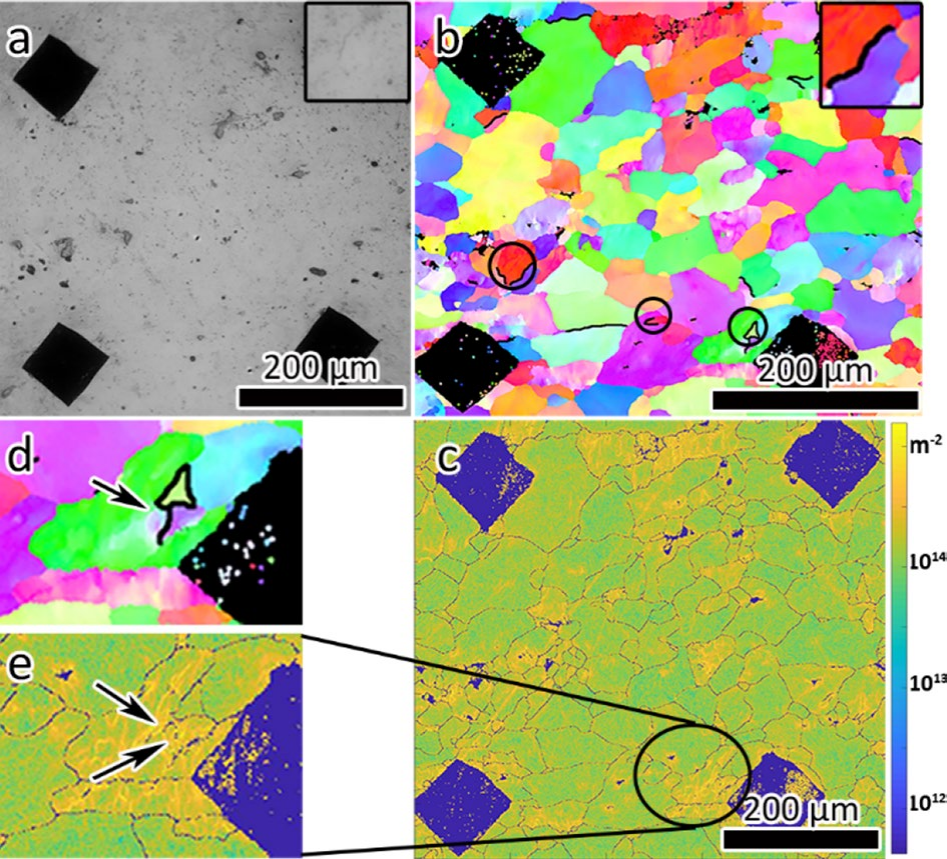
(**a**) Optical image showing the grain boundaries corroded after 600 s of acid exposure and (**b**) corroded grain boundaries highlighted by black lines in the IPF map. (**c**) Log-scale GND density map of the same region. (**d**), (**e**) Magnified images showing the corroded grain boundaries and the corresponding GND, respectively. The corroded grain boundary is highlighted in black and indicated by an arrow in (**d**). Arrows in (**e**) indicate regions of high GND density.

Figure 3a shows an optical image of the sample after 600 s exposure to phosphoric acid and an IPF map highlighting the identified corroded grain boundaries. The attacked boundaries were often associated with a single grain comprising of different grain boundaries with different grain boundary characteristics such as GND density and misorientation angle, as is highlighted in Fig. 3b. This suggests that grain characteristics and network effects, rather than grain boundary characteristics alone, can influence the susceptibility to intergranular attack. Out of a total of 952 unique grain boundaries in the characterized area, a total of 14 grain boundaries were attacked. The misorientation angles of the corroded boundaries ranged from 8° to 58°, suggesting that low angle grain boundaries are not necessarily resistant to β phase precipitation. Interestingly, it was found that there was a high dislocation density near the corroded low angle grain boundary (indicated by yellow in Fig. 3c, e), with a value of 6.4 × 10^14^ m^−2^, compared to the average GND density around all the grain boundaries of 2.3 × 10^14^ m^−2^. This grain boundary is highlighted in Fig. 3d, e. This indicates that dislocation density might have an important role in influencing the β precipitation, making them more susceptible to corrosion.

### Late stage microstructure effects and quantitative analysis

**Figure 4:**
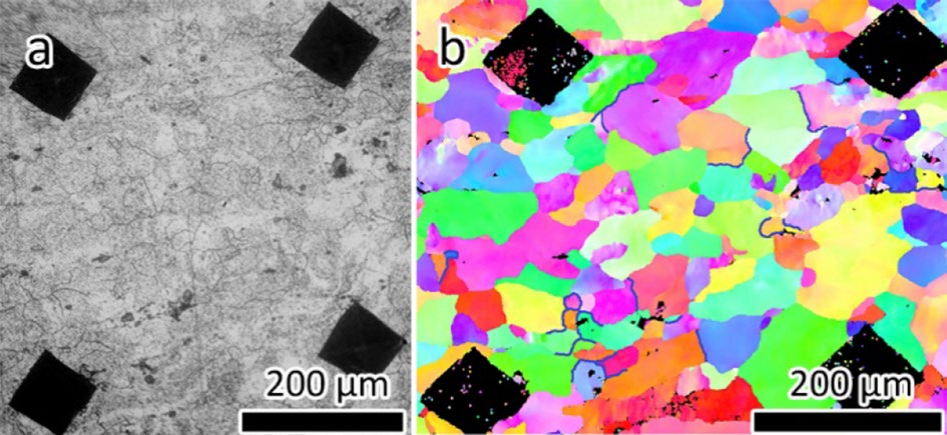
(**a**) Optical image showing the corroded microstructure after 2400 s exposure to H_3_PO_4_. (**b**) IPF map of imaged area with uncorroded grain boundaries highlighted by blue lines.

Figure 4 shows an optical image of the sample obtained at the end of the in situ optical microscopy experiment with an accompanying IPF map showing the uncorroded grain boundaries. 38 grain boundaries out of 952 were identified as uncorroded and marked in the IPF map (Fig. 4b). These uncorroded grain boundaries were often associated with a single grain or part of a grain boundary network, again suggesting that grain and network characteristics can play an important role in susceptibility to attack.

**Figure 5:**
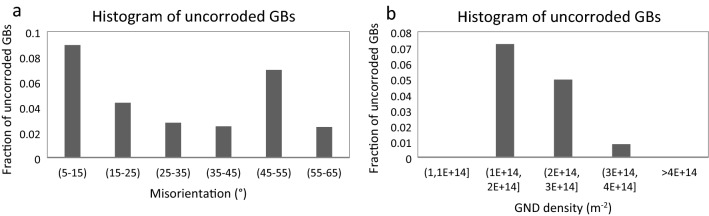
Normalized histogram plots of corroded grain boundaries with (**a**) misorientation angle and (**b**) GND density, after 2400 s of acid exposure (the average GND density around all the grain boundaries is 2.3 × 10^14^ m^−2^).

The identified uncorroded boundaries and microstructure information was used to plot the fraction of uncorroded boundaries as a function of misorientation angle and GND density. Figure 5a shows the histogram of the fraction of uncorroded grain boundaries after 2400 s as a function of grain boundary misorientation angle. As can be seen, almost all boundaries, regardless of the misorientation angle, are attacked by the phosphoric acid. The fraction of low angle uncorroded boundaries is slightly higher than the remainder of the boundaries, though the difference is fairly small, suggesting that misorientation angle is not the primary factor dictating grain boundary attack.

The histogram of GND density near the 38 uncorroded grain boundaries is shown in Fig. 5b. The average GND density of these uncorroded grain boundaries is 2.03 × 10^14^ m^−2^, which is slightly lower than the average value at all the grain boundaries. There is a clear correlation between the GND density and likelihood of attack, with grain boundaries with low GND density in the surrounding region being much less likely to be corroded. At high GND density, above 4 × 10^14^ m^−2^, all boundaries were attacked. This clear trend with GND density implies that this parameter does have a crucial role in determining the favorability of β precipitation and corrosion of grain boundaries. This once again suggests that misorientation angle alone cannot be taken as an indicator for precipitation and the interplay of other microstructural features like dislocation density affect precipitation. It should be noted that the dislocation density considered here is the local dislocation density, close to grain boundaries, not the global dislocation density.

To explore the statistical significance of our results, we performed a *t*-test on the data and based on this the *p*-value has been calculated. *p*-value was found to be 0.000675, suggesting that the results are statistically significant.

## Discussion

In the current study, in situ optical microscopy experiments were combined with EBSD analysis to determine the microstructure influences on β phase precipitation and intergranular corrosion. This combined approach facilitates the rapid characterization of a large number of grain boundaries (~ 1000 in this study), providing a statistical framework through which results from previous studies focused on a relatively small number of boundaries at high resolution can be understood. In most previous studies, TEM was employed to investigate the precipitation, providing direct resolution of the β precipitates, but limiting the number of boundaries that could be analyzed^17–23,42,43^. In addition, the majority of these studies focused on a single aspect of the microstructure such as misorientation angle or the influence of dislocation networks on precipitation kinetics^23,25,43^. The analysis conducted in the current study provides additional statistical support for these high-resolution studies and allows the relative effects of multiple aspects of the microstructure to be investigated simultaneously.

By the end of the 2400 s experiment, almost 96% of the grain boundaries exposed to phosphoric acid were found to be corroded. Both high and low angle boundaries were attacked (Fig. 5a). This shows that β precipitates form even at the low angle grain boundaries, which are often reported to be very resistant to precipitation and acid attack^17–19,42^. However, recent studies support the formation and existence of β particles on low angle grain boundaries^20,23^. The analysis in the present work suggests that the local GND density may account for the variations in β phase precipitation at different boundaries. This influence was seen both at the early stages of the corrosion experiment, where a low angle grain boundary with high surrounding GND density was attacked, and at the late stages of the experiment where boundaries with low surrounding GND density were shown to be the most resistant to attack (Figs. 3, 5). This higher susceptibility to β phase precipitation in regions of high dislocation density is likely due to the dislocations providing a diffusion pathway for Mg accumulation at the boundaries. It is well known that diffusivity through dislocation cores is higher in many systems than the diffusivity through the defect-free lattice^44^. Goswami et al. suggested that, based on a Zener-Hillert diffusion model, pipe diffusion of Mg through dislocation cores could account for thicker than expected β phase precipitates forming at grain boundaries^24^. Work done by Scotto D’Antuono et al. also made similar inferences. They demonstrated that cold rolling or straining an AA5xxx sample increases the kinetics of β growth^23^. This was again attributed to the enhanced diffusion of solute atoms via pipe diffusion due to the presence of dislocation bands close to the grain boundaries. There are few studies which suggest that increasing the dislocation densities via mechanical treatments, increases the resistance of these alloys to IGC^4,27^. However, it should be noted that these studies considered the impact of the global dislocation density (including the grain interiors), unlike the current study and previously discussed reports, where the effect of local dislocation networks were considered. Based on these observations, it is clear that local GND density plays an important role in dictating β phase precipitation in Al alloys.

It should be noted that the sample in this study was exposed to acid for 2400 s. If left further, the remaining uncorroded grain boundaries may have also been attacked. Further investigation is needed to determine whether the uncorroded grain boundaries lack β precipitates or if the state/size of the β precipitates on the boundaries delayed the attack. Also, we expect the indents to add some heterogeneity to the dislocation distribution. However, it is evident that the influence of both grain boundary characteristics and the state of the surrounding microstructure both play a role in determining susceptibility to IGC of individual boundaries.

## Conclusions

An approach combining in situ optical microscopy corrosion experiments with EBSD analysis was used to understand microstructure influences on the susceptibility to β precipitation and intergranular corrosion of individual grain boundaries. The experimental results support the following conclusions:

- Grain boundary misorientation angle alone is not a determining factor in whether or not a grain boundary is susceptible to attack. Multiple examples of low angle boundaries attacked at the earliest stages of exposure to an acidic environment and high angle boundaries remaining uncorroded over the course of the experiment were seen in the presented experiments.
- GND density in the immediate vicinity of a grain boundary showed the strongest correlation with intergranular attack, with boundaries with low surrounding GND density showing the highest resistance to corrosive attack.

These observed trends demonstrate the importance of considering other factors beyond grain boundary characteristics when determining the susceptibility of individual grain boundaries to β phase formation.

## Data Availability

The datasets generated during and /or analyzed during the current study cannot be shared at this time as the data also forms part of an ongoing study, but are available from the corresponding author on reasonable request.
